# Plagues, pandemics and epidemics in Irish history prior to COVID-19 (coronavirus): what can we learn?

**DOI:** 10.1017/ipm.2020.25

**Published:** 2020-04-15

**Authors:** B. D. Kelly

**Affiliations:** Department of Psychiatry, Trinity College Dublin, Trinity Centre for Health Sciences, Tallaght University Hospital, Dublin 24, Ireland

**Keywords:** Anxiety, coronavirus, COVID-19, epidemic, Ireland, mental disorders, pandemic, plague, psychiatry

## Abstract

**Objectives:**

This paper seeks to provide a brief overview of epidemics and pandemics in Irish history and to identify any lessons that might be useful in relation to psychiatry in the context of COVID-19.

**Methods:**

A review of selected key reports, papers and publications related to epidemics and pandemics in Irish history was conducted.

**Results:**

Viruses, epidemics and pandemics are recurring features of human history. Early Irish sources record a broad array of plagues, pandemics and epidemics including bubonic plague, typhus, cholera, dysentery and smallpox, as well as an alleged epidemic of insanity in the 19th century (that never truly occurred). Like the Spanish flu pandemic (1918–20), COVID-19 (a new coronavirus) presents both the challenge of the illness itself and the problems caused by the anxiety that the virus triggers. Managing this anxiety has always been a challenge, especially with the Spanish flu. People with mental illness had particularly poor outcomes with the Spanish flu, often related to the large, unhygienic mental hospitals in which so many were housed.

**Conclusions:**

Even today, a full century after the Spanish flu pandemic, people with mental illness remain at increased risk of poor physical health, so it is imperative that multi-disciplinary care continues during the current outbreak of COVID-19, despite the manifest difficulties involved. The histories of previous epidemics and pandemics clearly demonstrate that good communication and solidarity matter, now more than ever, especially for people with mental illness.

A new coronavirus, COVID-19, was identified in China in late December 2019 and quickly spread around the world (World Health Organization, 2020a). While the majority of people who contract the virus have a relatively mild, self-limiting illness, it can be fatal (Wang, 2020; Xu *et al.* 2020; Yang *et al.* 2020). Despite extensive public health measures, over 300 000 people were infected across 188 countries by March 2020 (Xiao & Torok, 2020). More than 13 000 had died. On 11 March, the World Health Organization (WHO) declared a pandemic. By early April, 1.5 million people were infected, and there had been over 85 000 deaths (WHO, 2020b).

Viruses, epidemics and pandemics are recurring features of human history (Camus, 1948; Garcia *et al.* 2015; Honigsbaum, 2019). The global influenza pandemic that lasted from 1918 to 1920, also known as the Spanish flu, is, perhaps, the best remembered pandemic of recent times (Foley, 2011; Honigsbaum, 2018; Milne, 2018). Public health responses to these events have changed considerably over time, but psychological responses appear more constant: mixtures of complacency, appropriate anxiety, excessive anxiety and panic (Kelly, 2020a).

These issues are especially to the fore in the current COVID-19 pandemic owing to unprecedented blanket media coverage of the outbreak (Cookson, 2020; Devlin & Boseley, 2020; *Economist*, 2020; Giuffrida, 2020; Kelly, 2020b). Some of the material published and shared, especially on social media, has been misleading and unhelpful: falsehoods, panic and hysteria will only weaken efforts to halt the virus (Kelly, 2020c).

Against this background, this paper seeks to provide a brief overview of epidemics and pandemics in Irish history and to identify any lessons that might be useful in relation to psychiatry in the context of COVID-19.

## Plagues, epidemics and other misfortunes in early Irish history

In 1856, Part V of the report of the *Census of Ireland for 1851* was published, co-authored by William Donnelly (Registrar General and Chief Commissioner), Dr William Wilde (Assistant Commissioner) and Henry Wilkie (Acting Secretary) (Donnelly *et al.* 1856). Dr William Wilde, now best known as father of the playwright, Oscar, was a prominent surgeon and author of numerous works on medicine, archaeology and folklore (Wilson, 1942; Coakley, 2016). Wilde was knighted in 1864 owing in large part to his work on the census (Froggatt, 1965; Froggatt, 2016; Geary, 2016).

The 1856 *Census* report that Wilde co-authored provided a fascinating summary of Ireland’s long history of outbreaks, epidemics and pandemics:

Having found that from the earliest period to which past chronicles refer down to the present time Ireland has suffered sometimes alone, and sometimes in common with Great Britain and the rest of Europe, from various epidemic pestilences, we collected and tabulated the circumstances attending them in the table attached to these introductory remarks. From an examination of this epitome of the most remarkable epidemic pestilences, as well as of the famines, epizootics, cosmical phenomena, and other circumstances, influencing, or supposed to influence mortality, we perceive that so far as the annals and records of the country afford information, Ireland has from the earliest period of its colonization to the present time been subjected to a series of dire calamities, affecting human life, arising either from causes originating within itself, or from its connexion with Great Britain and other parts of Europe (p.2).

The causes of these events were many and varied:

The literature of the times and the history of those early plagues, which devastated different parts of the world, show us that men usually endeavoured to account for such sudden outbursts of disease, either by the direct and miraculous interposition of Providence, or by some peculiar atmospheric condition, the manifestations of which were storms, hail, thunder, and lightning, unusual or sudden alterations of temperature, such as excessive heat, long continued drought, intense frost and snow, or great rains and inundations. Occasionally ‘signs and wonders’, eclipses of the sun or moon, comets, and certain prodigies and supernatural appearances in the heavens, are said to have been the forerunners of these disasters, affecting the animal or the vegetable world; and during the middle ages of the Christian era, the failure of crops, a murrain among cattle, or an epidemic affecting the human family, were often considered as punishments from heaven, for sacrilege or other crimes of that nature (p.2).

Wilde and colleagues showed a shrewd understanding of the epidemiology of infectious diseases that is just as relevant to COVID-19 as it was in ancient Ireland:

Many of the plagues from which this country suffered were continuations of those great waves of pestilence which had already passed (according to the general course of plagues) from the East, over the European continent, frequently carried along the track of human intercourse, by commercial dealings, or borne onward by hostile navies or invading armies; but others were more localised, were of domestic growth, and had their birth, and expended themselves within the circuit of this island - seldom spreading beyond its limits (p.2).

Exploring the early Irish literature, Wilde and colleagues found particularly ‘striking’ accounts of ‘the first and second outbreaks of the *Blefed*, or *Buidhe Connail* – the great Yellow Plague, which devastated Ireland in the sixth and seventh centuries’ (p.6). They went on to present a detailed ‘Table of Cosmical Phenomena, Epizootics, Famines and Pestilences in Ireland’ starting in ‘the Pagan or Pre-Christian Period’ and drawn, in large part, from the ‘Annals of the Four Masters’, chronicles of medieval Irish history complied in the 1630s. The first entry in the Table recounted the deaths of 5000 men and 4000 women in one week in ‘the place now called Tallaght near Dublin’ owing to ‘some sudden epidemic’ which was ‘the first recorded pestilence in Ireland’ (p. 41). The precise nature of this epidemic remains obscure.

The 1856 table compiled by Wilde and colleagues continues with an astonishing succession of epidemics, plagues, cosmic events and unfortunate occurrences, some of dubious veracity. The Annals of Innisfallen, for example, recorded that, in 569 AD there was ‘(*Bolgach*) smallpox amongst the people’ (p.47). Wilde and colleagues were sceptical: ‘This is the first notice of this disease in the Irish annals, and one of the earliest references thereto in any European authority. As it is not, however, verified in any other annal, the epidemic alluded to was probably the leprosy, then epidemic’.

The Annals of Boyle recorded ‘a great pestilence (*mortalitas magna*), that is the *Buidhe Chonnaill*’ in the (ominous) year of 666 (p.52). ‘Diarmaid and Blathmac, the two Kings of Ireland, died, as did Fechin of Fore, and many others thereof”. In 675, ‘there reigned a kind of great leprosie in Ireland this year, called the Pox, in Irish, *Bolgagh*’. This, according to Wilde and colleagues, ‘evidently was the small-pox, of which many distinguished persons died’.

Mysteries and tragedies abounded. In 678, ‘Lough Neagh was turned into blood’. The following year saw ‘universal pestilence’ as ‘England and Ireland were ravaged by it in 679; and in 680, during July, August and September, Rome was laid waste’. The year 695 saw ‘the cattle pestilence’ and there was ‘a great cow mortality’ in 707 (p.54). In 742, there was both ‘the *Bolgach* - small-pox’ and ‘dragons seen in the sky’ (p.55). In 814, there was ‘a great disease (*Scath mor*) and heavy sickness (*Tromghalar*)’ (p.59). Some entries in the Table are deeply obscure: in 847, for example, ‘Felym MacCriowhayn was overtaken by a great flux of the belly’. No further explanation is offered.

The Annals of Ulster recorded ‘a great Leprosy (*Clamthruscad mor*, scaly leprosy or mange) and running of blood (*Ruith fola*, Dysentery) upon the Gentiles of Dublin’ in 950 (p.63). Wilde and colleagues felt that this was an especially significant report:

These two notices would appear to apply to Syphilis, (and if so, to fix the introduction of it into Ireland), and the circumstances of the period tend to favour that idea. For some years before, there had been several Danish invasions, and immediately preceding the year 950 the Danes plundered a great part of Leinster, and took many captives - in one instance ‘upwards of three thousand persons’, so that the accession and spread of venereal affections is likely to have occurred at the time referred to.

## Bubonic plague, typhus, cholera, dysentery, smallpox and the Spanish flu

The ‘Black Death’, or Bubonic plague, raged in Ireland from 1348 to 1350, and it is likely that between a quarter and a third of the population died during the first outbreak, according to Joseph Robins (1995) in his invaluable book, *The Miasma: Epidemic and Panic in Nineteenth Century Ireland.* There were several more epidemics of plague which only declined across Europe in the mid-17th century, at which point typhus and dysentery became the chief threats. Smallpox, too, was major cause of death in 18th-century Ireland. An epidemic of typhus developed between 1816 and 1819 and was followed by cholera in the 1830s.

Wilde and colleagues described an earlier outbreak of typhus in 1225, based on the Annals of the Four Masters (p.77), and demonstrated clear links between illness, famine and conflict:

An oppressive malady (*Teidhm diofhulaing*, irresistible pestilence) raged in the province of Connaught at this time; it was a heavy, burning sickness (*Treabhlaid Tromteasaiglithi*), which left the large towns desolate, without a single survivor’. This hot, heavy, death sickness, not sudden as the *tamh*, was probably our Irish typhus, which succeeded to the war and famine which desolated large portions of Ireland at this period; so that ‘woeful was the misfortune which God permitted to fall upon the best province in Ireland at that time for the young warriors did not spare each other, but preyed and plundered each other to the utmost of their power. Women and children, the feeble, and the lowly poor, perished by cold and famine in this war.

These themes were again in evidence during the Great Irish Famine of 1845 to 1849 which saw epidemics of typhus, cholera, dysentery and smallpox, as well as ‘mental disease’ which ‘bore its part in the list of calamities, upon which it is our duty to report’ (p.254). ‘The receptions into Lunatic Asylums…greatly increased and the deaths from insanity [became] greater from 1847 to 1850’ as part of ‘that great calamity which befel this country during the years of famine and pestilence’.

The 19th century was also the great asylum-building era in Ireland, when large public institutions were erected for the mentally ill right across the country (Kelly, 2016). The new asylums were immediately overcrowded, fuelling fevered speculation that the rate of mental illness was increasing uncontrollably in Ireland (and elsewhere). It is now clear that Ireland never had an epidemic of mental illness (Brennan, 2014), but, at the time, this panic was slow to subside, fuelled by the ever-expanding institutions, ill-advised mental health legislation and alarmist reports in professional and popular media.

The early 20th century also saw the most dramatic pandemic of recent history, the global influenza pandemic or ‘Spanish flu’ that killed at least 50 million people worldwide between the spring of 1918 and the winter of 1919 (Honigsbaum, 2018). In Ireland, the flu infected almost 800 000 people and more than 20 000 died (Foley, 2011). The pandemic had enormous effects across Irish society (Milne, 2018). The large, overcrowded asylums were hit especially hard: a fifth of all patients in Belfast asylum died of the flu, and one patient in every seven in the asylums in Kilkenny, Castlebar, Maryborough and Armagh fell victim (Robins, 1986; Kelly, 2016).

Tuberculosis was another persistent problem in Ireland through much of the 20th century, becoming the leading cause of death in Irish children in the 1930s (Ferry, 2019). It, too, was especially problematic in the mental hospitals (Kelly, 2016). The tuberculosis epidemic did not end until the late 1950s as a result of testing, vaccination and effective antibiotics. The advent of COVID-19 in early 2020 prompted many media comparisons of the new coronavirus virus with not only the Spanish flu and tuberculosis but also HIV/AIDS in the 1980s, severe acute respiratory syndrome in 2003 (SARS, which caused no deaths in Ireland) and the influenza pandemic of 2009 (‘swine flu’, which caused 27 deaths in Ireland) (Bielenberg, 2020). Such comparisons are interesting and useful provided they are accompanied by an awareness that the medical, social, political and economic circumstances of each outbreak can be quite different (Peckham, 2020). As a result, each outbreak, epidemic and pandemic is, in many ways, unique.

## What can we learn from plagues, pandemics and epidemics in Irish history prior to COVID-19?

A century after the Spanish flu pandemic, COVID-19 emerged in China during the last week of December 2019 (WHO, 2020a). As was the case in 1918, the new virus presents two clear challenges to the world: the illness caused by the virus itself and the anxiety and panic that it triggers around the globe (Cullen *et al.* 2020; Kelly, 2020a; Milne, 2020). The ubiquity of speculative and false information about COVID-19 presents particular problems to public understanding and rational management of the outbreak (Cookson, 2020; Harford, 2020).

This problem is not new. Exaggerations, rumours, myths and falsehoods also abounded during the Spanish flu pandemic, causing confusion, distress and a great deal of panic (Foley, 2011). Today, social media are especially prone to such issues (Lanier, 2018). In February 2020, the WHO took specific steps to counter the inaccuracies and conspiracy theories that were spreading in both social and conventional media (*Lancet*, 2020a; Zarocostas, 2020). Various medical journals made reliable information available for healthcare professionals (*Lancet*, 2020b; Razai *et al.* 2020). These steps are important. The experience of the Spanish flu clearly demonstrates the power of myths to persist and the urgent requirement for calm, factual information to counteract unhelpful panic.

Another key lesson from the Spanish flu is that pandemics have profound and lasting psychological effects on the population. Milne (2018) writes about the emotional effects of the Spanish flu, exploring the losses that people suffered and the psychological traumas that lasted, in many cases, for a lifetime. It is important to recognise these long-term psychological effects during all phases of the pandemic and to offer psychological interventions to people directly affected by the virus (Duan & Zhu, 2020).

Regrettably, some of the public health measures designed to control transmission, such as isolation and quarantine, can have negative effects on psychological well-being. While these measures are effective for controlling the spread (Hellewell *et al.* 2020), strategies such as quarantine are also associated with specific stressors, such as fear of infection, frustration, boredom and problems stemming from inadequate supplies and insufficient information (Brooks *et al.* 2020). After quarantine, there can be concerns about finances and stigma, among other matters. These negative effects can be ameliorated by terminating quarantine as soon as possible, providing adequate information and supplies, reducing boredom and improving communication.

As was the case during the Spanish flu pandemic (Milne, 2018), many children’s lives have been deeply disrupted by COVID-19. The problems are particularly acute in children who are largely confined to home owing to school closures, family illness or physical distancing. In this setting, it important that children are guided about online learning, but are not overburdened with work; that parents work with schools to ensure healthy lifestyles and that good diet, personal hygiene and sleep habits are encouraged (Wang *et al.* 2020). It can also be useful for parents to have direct conversations with children about the outbreak in order to alleviate their anxieties.

One of the greatest psychological challenges of a prolonged pandemic is learning to live with uncertainty not only about the virus (Vetter *et al.* 2020) but also about the outbreak’s long-term effect on our societies and our way of life (Harari, 2020; Jenkins, 2020; Mance, 2020; Naughton, 2020; Ord, 2020). Both the epidemics during the Great Irish Famine and the Spanish flu had profound effects on Irish society (Robins, 1995; Foley, 2011; Milne, 2018). In this context, it is important, in the current pandemic, to find time to reflect on one’s thoughts and emotions about such uncertainty (Chödrön, 2016; Kelly, 2020a), to care for one’s physical health, giving particular priority to sleep (Walker, 2017) and to follow the mental health guidance provided by the WHO (2020c), US Centers for Disease Control and Prevention (2020) and US Substance Abuse and Mental Health Services Administration (2014).

From the perspective of psychiatry, one of the other lessons from the epidemics and pandemics of the past is that not everyone suffers equally: the poor suffered disproportionately during the epidemics of the Great Irish Famine, and people with mental illness were especially vulnerable to the Spanish flu. The reason at that time was that so many of the mentally ill were confined in large, unhygienic mental hospitals through which the flu spread at high speed (Kelly, 2016). Today, mental health care is, for the most part, based in the community, but there is still every reason to believe that people with mental illness are at increased risk.

Even prior to COVID-19, it was known that people with mental illness still have a lower life expectancy and poorer physical health than the general population (Rodgers *et al.* 2018). This places the mentally ill at increased risk in a pandemic, especially for community transmission of the virus. This also underlines the importance of continued multi-disciplinary care during the outbreak, despite the inevitable difficulties involved in delivering such care.

Looking to the future more broadly, there is moderately strong, long-standing evidence that prenatal infection with viruses, such as influenza, might increase the risk of schizophrenia in offspring in later life (Kępińska *et al.* 2020). This possibility suggests a need for longitudinal follow-up studies of babies born during or soon after the current COVID-19 pandemic, to assess their developmental indices as they grow and their risk of various mental illnesses in later life. More immediately, this line of research emphasises the importance of public health measures to control transmission of COVID-19 in the first place, including rapid testing, isolation of cases and general physical distancing.

All of these measures require broad-based community support in order to be effective. In early March 2020, the WHO Director-General (2020) emphasised the need for ‘solidarity’ in the face of COVID-19. For all of us, and for people with mental illness in particular, this ‘solidarity’ is essential if we are to learn the lessons of the epidemics and pandemics of Irish history and minimise the impact of COVID-19 on physical and mental health today.
