# Plagues, pandemics and epidemics in Irish history prior to Covid-19 (coronavirus): What can we learn? - Corrigendum

**DOI:** 10.1017/ipm.2020.97

**Published:** 2020-12

**Authors:** Brendan D. Kelly

In this paper, the sentence ‘In Ireland, the flu infected almost 800 000 people and more than 20 000 died (Foley, 2011).’ should read: ‘In Ireland, the flu infected almost 800 000 people and more than 20 000 died (Beiner *et al.* 2009; Milne, 2018).’
